# Volume Phase Transition in Gels: Its Discovery and Development

**DOI:** 10.3390/gels6030022

**Published:** 2020-07-31

**Authors:** Karel Dušek, Miroslava Dušková-Smrčková

**Affiliations:** Institute of Macromolecular Chemistry, Czech Academy of Sciences, Heyrovského náměstí 2, 162 06 Prague 6, Czech Republic; dusek@imc.cas.cz

**Keywords:** volume phase transition, phase separation, gel, stimuli-responsive, swelling, polymer network, cross-linking, Gibbs energy

## Abstract

The history of volume phase transition of responsive gels from its theoretical prediction to experimental discovery was described and the major role of mixing Gibbs energy function in theoretical models was stressed. For detailed analysis and fine tuning of the volume phase transition, the generalized Flory–Huggins model with concentration and temperature dependent interaction function coupled with Maxwell construction as a tool is very suitable. Application of expansive stresses can uncover the potential of various swelling gels for volume phase transition. Experimentally, the abrupt, equilibrium-controlled phase transition is often hard to achieve due to passage of gel through states of mechanical instability and slow relaxation processes in macroscopic objects.

## 1. Introduction

The volume phase transition (VPT) in gels is characterized by an “abrupt” (discontinuous) change in the degree of swelling and by the possibility of coexistence of two gel phases differing in the degree of swelling. It is a special kind of phase equilibria pertaining to swollen cross-linked systems. The idea of the existence of volume phase transition originated in the consideration of phase separation during network formation.

In early 1960s, macroporous cross-linked ion exchange resins became interesting because of their combined ion exchange and selective adsorption properties [1]. Macroporous matrices were prepared by cross-linking free-radical copolymerization in presence of additives—low-molecular weight diluents or of a soluble polymer. The experimental experience gathered at Permutit, Ltd. in U.K. (John Millar’s group) was published in a series of articles [2,3,4,5]. In parallel, some practically oriented research was going on at Rohm and Haas, Inc. in the U.S. (R. Kunin), but quite broad research was started on in one of the Czechoslovak research centers. Their work was summarized later in a review [6]. It is interesting to note that the research covered not only macroporous materials, but also micro- to mesoporous highly cross-linked polymer matrices of high specific pore surface which nowadays belong to the group of metal–organic frameworks—a hit of first and second decades of the 21st century because of their interesting application properties—storing of gases (hydrogen), specific catalysis, etc. (cf., e.g., [7]). At the same time, the research on ion-exchange matrices addressed a variety of separation materials for just emerging gel permeation chromatography [7].

The findings of pore formation were initially interpreted empirically, but we had a strong desire to embed them into the framework of the polymer theory at that time represented by the P. J. Flory’s Principles of Polymer Chemistry “bible” [8]. Formation of heterogeneous structures was considered thermodynamically as phase separation and the incipience of phase separation was identified with the moment when the cross-link density (a function of the polymerization conversion) reached such value that the maximum degree of swelling in the existing liquid (diluent + monomer) reached its actual value in the system ($\phi_{2}=\phi_{2}^{0}$) [9,10,11]. Phase separation was enhanced by increasing dilution, increasing polymer-diluent interaction parameter *χ*, increasing degree of cross-linking and increasing molar volume of the diluent. For the first time it was made clear why good solvents and soluble polymers identical in chemical composition with network chains are so effective in phase separation. Further studies of phase separation in gels explained when and why in some cases phase separation occurs in micro or macro forms [12,13]. A number of approximations were adopted concerning the evolution of structure, role of sol fraction, system dispersity, multicomponent interactions, etc.

In the course of analysis of various factors on phase separation, cases were encountered when the dependence of the chemical potential of the diluent ($\Delta \mu_{1}/RT$) or its Maxwell construction form ($\Delta \mu_{1}/RT\phi_{2}^{2}$) on the volume fraction of polymer ($\phi_{2}$) passed through two extremes corresponding to three roots satisfying the condition $\Delta \mu_{1}=0$ for pure diluent. This special feature indicated the possibility of a three-phase equilibrium between two gel phases differing in the degree of swelling and satisfying the condition

(1) $$\mu_{1}^{\prime }=\mu_{1}^{\prime \prime }=0;\;\mu_{2}^{\prime }=\mu_{2}^{\prime \prime }$$

where $\mu_{2}(\phi_{2})$ was calculated from the Gibbs–Duhem equation or just from $\Delta G_{\mathrm{sw}}$ showing two minima. For the Flory–Huggins mixing and the Gaussian network elasticity case, such treatment [14] resulted in phase diagram showing an abrupt (discontinuous) change of the degree of swelling with temperature or other stimuli, the position and width of which was determined by fixed values of the concentration of elastically active network chains, degree of dilution and Flory–Huggins interaction parameter.

At that time, such prediction was quite puzzling because nothing like that was observed experimentally before and the picture of a possible coexistence of two phases differing in the degree of swelling within a macroscopic piece of covalently cross-linked gel seemed absurd. Moreover, from theoretical models of phase separation and experimental experience, preparation of a network combining the necessary ranges of cross-link density, dilution and polymer-solvent interaction seemed difficult because of a possible liquid–gel phase separation was likely to occur. However, it is necessary to note that our considerations were based on then available experience with weakly polar non-ionizable polymers and solvents.

The experimental evidence of such kind of volume phase transition was supplied by Toyoichi Tanaka and collaborators (cf., e.g., refs. [15,16,17]) who recognized the important role of specific interactions involving hydrophilic sites and water. Ionized groups fixed to the network chains of the gel were especially effective because they were capable to provide highly hydrated counter ions. Theoretically, it meant addition of a negative term to the chemical potential expression causing transformation of a monotonous dependence of the chemical potential of the solvent on $\phi_{2}$ into such dependence showing two extremes necessary for phase transition to occur. The theoretical interpretation of the experiment was made along similar lines as that in 1968, but fully independently. Toyoichi Tanaka’s papers had started a great interest in the phenomenon of volume phase transition first in Japan and later worldwide. The number of papers on this phenomenon published so far is estimated to have reached the order of 10^4^. However, not in all papers the phenomenon of phase transition is well understood and not all interpretations of experimentally observed steep changes of the swelling degree are well substantiated. The state of art at the given time aimed mainly on VPT interpretation and modeling [17,18,19,20,21,22,23,24,25]. The change of temperature has been the primary stimulus to induce volume phase transition, but the transition can be induced by any stimulus by which the $\Delta G_{\mathrm{sw}}$ and $\Delta \mu_{1}$ are changed: either such as degree of ionization of ionizable groups, mechanical strain, solvent exchange or indirectly such as by light (conformational changes), electric fields, etc. In this short overview, we want to emphasize two aspects: (a) how to use a simple theoretical analysis to help designing and synthesizing gels that potentially show VPT and (b) the forms of VPT observed experimentally. Concerning the aspect (a) we will show the usefulness of observation of the changes using Maxwell construction and using generalized interaction function in the Flory–Huggins theory.

## 2. Mean-Field Description of Volume Phase Transition

Let us review the basic mean-field theory in order to understand the role of basic parameters in VPT. The swelling equilibrium is characterized by change of the Gibbs energy $\Delta G_{\mathrm{sw}}$ considered as additive contributions due to mixing of the cross-linked polymer with the solvent, $\Delta G_{\mathrm{mix}}$, and due to deformation of elastically active network chains, $\Delta G_{\mathrm{el}}$. In the case of presence of ionizable groups, $\Delta G_{\mathrm{sw}}$ is contributed by the Donnan effect (presence of counter-ions and possibly co-ions), $\Delta G_{\mathrm{ion}}$, and in some cases by electrostatic repulsion between fixed charges of the same sign, $\Delta G_{\mathrm{elstat}}$, cf., e.g., ref. [26,27],

(2) $$\Delta G_{\mathrm{sw}}=\Delta G_{\mathrm{mix}}+\Delta G_{\mathrm{el}}+\Delta G_{\mathrm{ion}}+\Delta G_{\mathrm{elstat}}$$

In this overview due to its limited extent, we will consider non-ionized networks only, i.e., $\Delta G_{\mathrm{sw}}=\Delta G_{\mathrm{mix}}+\Delta G_{\mathrm{el}}$. This is not because the ionization factor is less important, on contrary, introduction of $\Delta G_{\mathrm{ion}}$ can induce VPT in many networks with interesting backbone chains.

## 3. The Mixing Contribution

Over the past century, polymer-solvent mixing was one of topical disciplines of macromolecular science. In addition, it plays the most important role in VPT phenomena. Since the Flory–Huggins simple lattice model with purely enthalpic interaction term, the theoretical models advanced in several directions. Only a few representative literature sources are added here to characterize the main directions. It was first of all improvement of the lattice model taking into account more realistically the topology of the chain structures starting from the works of Guggenheim to Dudowicz and Fried [28,29,30,31,32] and consideration of the free-volume and volume changes on mixing [33,34,35]. In the double lattice model of Oh and Bae [35] conventional weak physical interactions in polymer solutions were described by the primary lattice, while a secondary lattice was introduced as a perturbation to account for oriented interactions. Perturbed-chain statistical associating fluid theory (PC SAFT), a hard core chain model, is suitable to deal with systems containing polymers and associating fluids [35,36]. Other theories view solutions with strong interactions as reversible, temporary clusters (networks) [37,38,39]. In addition, the fact has been considered that equilibrium (at “infinite” time) is hardly to reach experimentally in strongly interacting systems [40]. All these theories mentioned above work with physically defined models described by physically meaningful parameters, although their values are often not available and difficult to assess.

On contrary the generalized Flory–Huggins model is based on the very simple mixing entropy of non-interacting chains (combinatorial entropy) and an enthalpic-entropic excess function in which all deviations of a real system are absorbed. This excess function is expressed through the concentration (and temperature) dependent interaction function [40] $g(\phi_{2})$ which takes into consideration interactions of higher than binary orders. This concentration dependence of $g(\phi_{2})$ can also be expressed as a power series of polymer volume fraction $\phi_{2}$. In fact, the results of the various theories mentioned in the preceding paragraph can be approximated by expansion into such power series with specific dependences of the coefficients on temperature.

For cross-linked systems, it has been suggested that the combinatorial part of $\Delta G_{\mathrm{mix}}$ refers to the loss of entropy due to coupling of segments into clusters of functionality *f* [41,42,43] because there is no logical reason to reduce $\Delta G_{\mathrm{mix}}$ for networks to solutions of linear polymers of infinite molecular weight. Within this concept, the extensive form of $\Delta G_{\mathrm{sw}}$ for Flory–Huggins lattice model reads

(3) $$\Delta G_{\mathrm{mix},n}=k_{B}T(N_{1}\ln\phi_{1}+\Xi_{e}\ln\phi_{2}+g_{N}(\phi_{2})N_{1}\phi_{2})$$

where subscript n denotes a network, $N_{1}$ is number of moles of solvent, $\phi_{1}$ and $\phi_{2}$ are volume fractions of solvent and polymer, respectively, $\Xi_{e}$ is the cycle rank of elastically active network chains, EANCs; it holds $\Xi_{e}=((f_{e}-2)/f_{e})N_{e}$ where *f*_e_ is the number–average number of EANCs issuing from an elastically active crosslink,$N_{e}$ is the number of elastically active chains; $g_{N}(\phi_{2})$ is the interaction function. The two first terms of eq. 3 can be considered as the combinatorial part of $\Delta G_{\mathrm{mix},n}$ for a network. Proceeding to the chemical potential of the solvent in the usual way, one gets

(4) $$\frac{\Delta \mu_{1,\mathrm{mix},n}}{RT}=\left(\frac{1}{m_{1}}\ln\phi_{1}+\frac{\phi_{2}}{m_{1}}+\frac{f_{e}-2}{f_{e}}\nu_{e}V_{\mathrm{mol},1}\phi_{2}+\left(g(\phi_{2})-\frac{\partial g(\phi_{2})}{\partial \phi_{2}}\phi_{1}\right)\phi_{2}^{2}\right)$$

where $V_{\mathrm{mol},1}$ is the volume of lattice site, $\nu_{e}$ is the concentration of elastically active network chains per volume, and $m_{1}$ is the ratio of molar volumes of solvents to volume of lattice site. Such rationalization makes it possible to compare interactions for solvents of various sizes because the interaction energies are related to the same volume/mass of polymer.

Equation (4) shows us why the mixing contribution is so important for reaching the VPT behavior of polymer gels. Besides the size of the solvent molecule relative to the size of polymer segment, it is the interaction function, especially its concentration and temperature dependences. Even for uncross-linked polymers, its special forms can produce not only UCST and LCST phase diagrams, but also closed-loop or, hour–glass shapes [44] where the immiscibility gap increases with increasing molecular weight. The maximum/minimum temperature for solutions of polymers of molecular weight approaching infinity is reached at polymer concentrations approaching zero (zero critical concentration systems). Yet, there exist a special group of polymer-solvent system for which the critical temperature is reached not at zero concentration, but at a concentration quite far from zero. These systems were called systems with “off-zero critical concentration” (OZCC). Such behavior was first observed experimentally [45,46] and later generalized referring to their importance for existence of VPT [47,48]. The detailed analysis [48] has shown that there exist several classes (within certain ranges of values of coefficients of power series expansion of $g(\phi_{2})$) showing one, two or three OZCCs. Such analysis was then applied to several experimental gel systems showing VPT such as PNIPAm, [49,50] or poly(vinyl methyl ether) [51,52]. The gels used in these experimental studies are lightly cross-linked mainly in order to prevent them from transforming into liquids when the critical value of stimulus is surpassed. For this class of VPT gels, the effect cross-links on $\Delta \mu_{1,\mathrm{sw}}$ is minor but needed.

## 4. The Elastic Contribution and Swelling Change of the Chemical Potential

In cross-linked gels, the osmotic pressure generated by mixing is opposed by the force generated due to stretching of network chains and the respective contribution to the Gibbs energy (at low external pressures equals to the Helmholtz energy) for a network (subscript n) can be expressed as

(5) $$\Delta G_{\mathrm{el},n}\approx \Delta F_{\mathrm{el},n}=\Delta F_{\mathrm{el}}-RTX_{e}\ln\left(V/V_{0}\right)$$

where $X_{e}$ is either $N_{e}$ or $\Xi_{e}$ (eq. 3) and depends on whether phantom or affine behavior is considered and $V/V_{0}$ is the swollen volume relative to the reference volume; $\Delta F_{\mathrm{el}}$ refers to elastically active network chains. Several rubber elasticity theories are available. The best known and widely used is based on model of network of Gaussian chains (cf., e.g., ref. [53]); for highly swollen or stretched gels, models respecting the finite extensibility of network chains are required. One of such equations was used for the analysis of the effect of strain in ref. [43] and a choice of them can be found in ref. [41]. Thus, for the Gaussian networks

(6) $$\Delta F_{\mathrm{el}}/kT=\frac{\Xi_{e}}{2}(\lambda_{x}^{2}+\lambda_{y}^{2}+\lambda_{z}^{2}-3)\;\mathrm{and}\;\Delta F_{\mathrm{el}}/kT=\frac{N_{e}}{2}(\lambda_{x}^{2}+\lambda_{y}^{2}+\lambda_{z}^{2}-3)$$

for phantom network (The combination the volumeless phantom model for elasticity with volume occupancy determined mixing model is unphysical.) and affine network, respectively, where *λ*_x_, *λ*_y_ and *λ*_z_ are the deformation ratios along the x, y and z axes related to the isotropic reference dimensions. This gives, for affine network

(7) $${\left(\Delta \mu_{1}\right)}_{\mathrm{el}}/RT=V_{1m}\nu_{e}\phi_{2}^{1/3}{\left(\phi_{2}^{0}\right)}^{2/3}$$

This gives for equilibrium swelling of a covalently cross-linked gel in a solvent of activity *a*_1_

(8) $$\frac{1}{m_{1}}\ln\phi_{1}+\frac{\phi_{2}}{m_{1}}+\frac{f_{e}-2}{f_{e}}\nu_{e}V_{\mathrm{mol},\mathrm{ls}}\phi_{2}+\left(g(\phi_{2})-\frac{\partial g(\phi_{2})}{\partial \phi_{2}}\phi_{1}\right)\phi_{2}^{2}+\nu_{e}V_{\mathrm{mol},\mathrm{ls}}\left(\phi_{2}^{1/3}{\left(\phi_{2}^{0}\right)}^{2/3}-\phi_{2}\right)=\ln a_{1}$$

The swelling degree and swelling behavior are determined by

- $m_{1}$ volume of solvent molecule with respect to the volume of lattice site: increasing causes decreasing of swelling degree;
- $f_{e}$ effective functionality of an elastically active crosslink which can vary between 3 and chemical functionality for perfect network: $f_{e}$ increasing causes decreasing of degree of swelling;
- $\nu_{e}$ concentration of elastically active network chains; with $\nu_{e}$ increasing swelling degree decreases;
- $g(\phi_{2})$ the interaction function and its concentration dependence: increasing $g(\phi_{2})$ usually contributes positively to $\Delta \mu_{1}$ (decreases the region of thermodynamic stability). The concentration dependence can cause VPT behavior;
- $\phi_{2}^{0}=1-\phi_{1}^{0}$, $\phi_{1}^{0}$ is the volume fraction of diluent present during network formation: increasing $\phi_{1}^{0}$ increases degree of swelling; too high $\phi_{1}^{0}$ may cause reaction induced phase separation during formation of the network.

### Swelling Under Constraint

The equilibrium swelling degree is dependent on deformation. This dependence is sometimes utilized in application, in other cases, it is rather a complicating factor. Extensive literature exists on the effect of deformation on swelling of gels including those showing VPT [36,54,55,56,57,58]. Sometimes, this dependence can serve to make the transition observable for systems that do not show the transition at free swelling. To use uniaxial elongation to make the transition visible for a UCST system was already suggested in the first paper on VPT [14]. Subjecting gels to strain was suggested as a way of uncovering the potential to show VPT behavior of various polymer-solvent system [43]. Indeed, many systems seem to hide this potential and a small chemical modification could shift the abrupt change of the degree of swelling to be observable at free swelling conditions. The elastic Gibbs energy contribution is expressed as a function of deformation ratios along the x, y, z-axes

(9) $$\Delta G_{\mathrm{el},n}\approx \Delta F_{\mathrm{el},n}=\Delta F_{\mathrm{el}}(\lambda_{x},\lambda_{y},\lambda_{z})-RTN_{e}\ln(\lambda_{x}\lambda_{y}\lambda_{z})$$

Generally, expansive strains decrease the chemical potential and increase the degree of swelling while compressive modes act in the opposite direction. Thus, the researcher has several handles available through which she/he can finely tune the parameters by changing the conditions of gel synthesis

- by changing molar volume of solvent molecule;
- somewhat by changing functionality of cross-links;
- by changing cross-link density (i.e., the concentration of EANCs);
- by changing the interaction function and its concentration dependence through (minor); changes of the chemical composition of the gel polymer (e.g., copolymerization) or small change in the solvent structure;
- by increasing or decreasing dilution during network formation.

## 5. Tools for Fine Tuning of Volume Phase Transition

**Figure 1:**
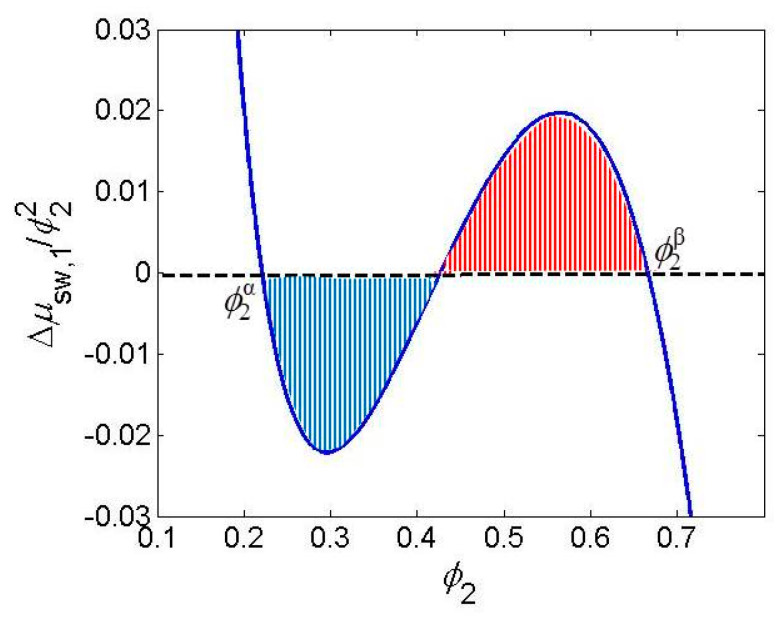
Maxwell construction.

Fine tuning of the gel system to show VPT behavior at conditions of observation requires a piece of chemical and physicochemical sense (intuition) and a means by which the effect of such structural change is visualized. Intuition means to find easy modification at synthesis to alter interactions (copolymerization, grafting, monomer bulkiness, number of H bonds donor or acceptor sites, etc.). As visualization means, the Maxwell construction was offered [43] which was already used by Shibayama and Tanaka [17] for finding of composition of coexisting phases. The condition for existence of the VPT is the existence of two extremes on the $\Delta \mu_{1}\;\mathrm{vs}.\;\phi_{2}$ dependence (van der Waals loop). According to the Gibbs–Duhem equation, the equality of chemical potential of the solvent is reached at two non-zero concentrations if in the plot of $(\Delta \mu_{1}/\phi_{2}^{2})$ vs. $\phi_{2}$ (Figure 1, the integral *I* = 0.

(10) $$I=\int_{\phi_{2}^{\beta }}^{\phi_{2}^{\alpha }}(\Delta \mu_{1}/\phi_{2}^{2})d\phi_{2}=0$$

**Figure 2:**
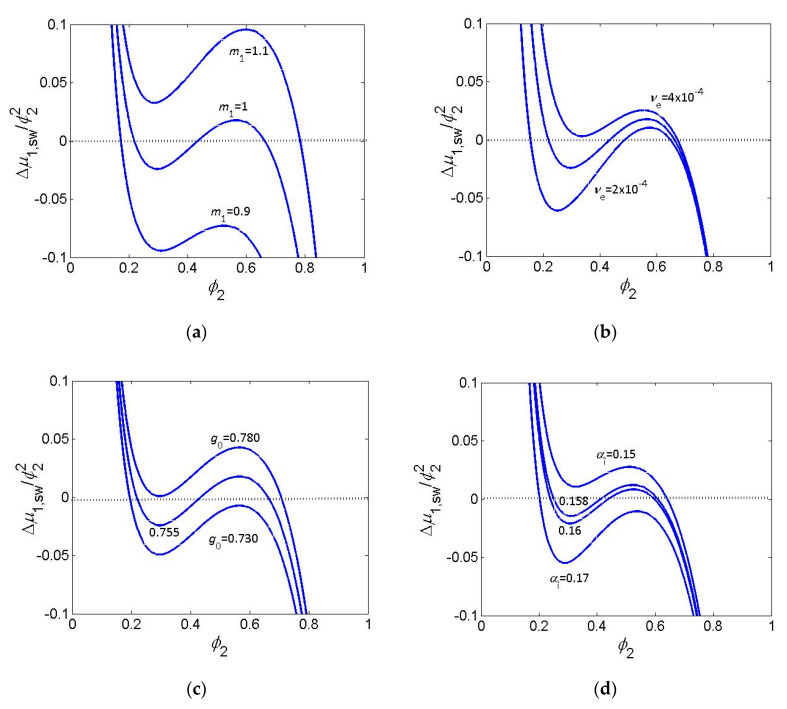
Maxwell plot. Effect of changing values of parameters on the shape and position of the van der Waals loop. (**a**) Ratio of volumes of solvent molecule to polymer segment for $\nu_{e}=3\times 10^{-4}{\mathrm{mol}/\mathrm{cm}}^{3}$, $g_{0}=0.755$, $g_{1}=0.600$; (**b**) concentration of EANCs in mol/cm^3^ for $m_{1}=1$, $g_{0}=0.755$, $g_{1}=0.600$; (**c**) constant *g*_0_ in interaction function $g(\phi_{2})$ for $m_{1}=1$, $\nu_{e}=3\times 10^{-4}{\mathrm{mol}/\mathrm{cm}}^{3}$, $g_{1}=0.600$; (**d**) degree of ionization *α*_i,_ for $m_{1}=1$, $\nu_{e}=1\times 10^{-3}{\mathrm{mol}/\mathrm{cm}}^{3}$, $g_{0}=1.0$, $g_{1}=0.400$. For all dependences $f_{e}=4$, $V_{\mathrm{ls}}=100$.

The effect of some of the parameters discussed above on the shape and position of the vdW loop is illustrated by Figure 2a–d.

The change of parameters makes the vdW loop more or less pronounced and shifts the dependence along the ordinate axis with the aim to fulfil the condition *I* = 0 for the given solvent activity, *a*_1_, for free swelling *a*_1_ = 1. For comparison, the effect of ionization degree of a polyelectrolyte gel is included. One can see that very delicate changes of parameters are necessary to accommodate the VPT behavior to equilibrium condition.

**Figure 3:**
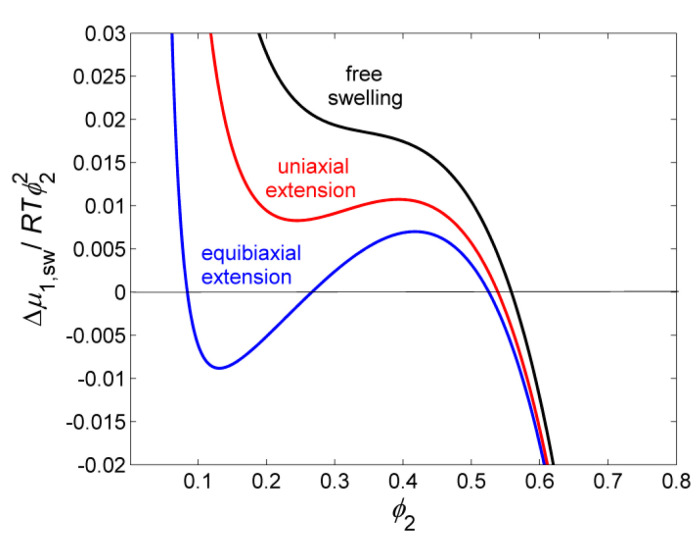
Illustration of the effect of various modes of strain on shape and position of dependences of the Maxwell plot.

Mechanical strains act similarly, as it is shown in Figure 3. Expansive strains shift the plots to lower values $(\Delta \mu_{1}/\phi_{2}^{2})$ and make the vdW deeper, compressive stress act oppositely; sometimes, the vdW loop disappears completely.

**Figure 4:**
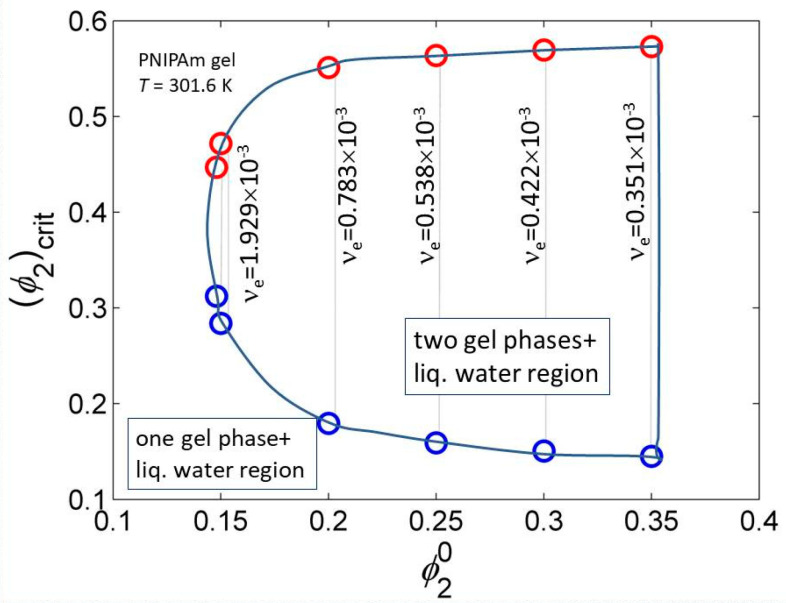
Region where two PNIPAm gel phases are in equilibrium with liquid water at *T* = 301.6 K and allowed combinations of $\nu_{e}\;\mathrm{and}\;\phi_{2}^{0}$. Interaction function parameters calculated using data from ref. [50].

The equality (10) is also used for finding compositions of conjugated phases and for construction of phase diagrams. Choosing, for instance, the transition temperature that determines the value of the interaction function of a particular system, (and *m*_1_) while the pairs of the values $\nu_{e}$ and $\phi_{2}^{0}$ are fixed; an example for PNIPAm–water gel is shown in Figure 4.

For a fixed transition temperature (i.e., value of *g*), the two gel-phases region is limited to a narrow choice of cross-link densities and dilutions. Although for PNIPAm and similar gels their LCST given by $g(T,\phi_{2})$ is dominating, the dependences on $\nu_{e}$ and $\phi_{2}^{0}$ are important for fine tuning.

In the literature, large data exist on chemically different systems both non-ionic (PNIPAm based gels are in lead) and ionic ones. Volume changes are interpreted in terms of equilibria analogous to those discussed here. In addition to temperature, changes in composition of mixed solvents are used as VPT stimuli. Using the single-liquid approximation for interpretation of data are very inaccurate since this approach neglects preferential sorption and excludes the possible four-phase equilibria (two gel and two liquid phases).

## 6. Equilibrium Theory vs. Experimental Observations

From early times of VPT, it was realized and observed that the experiment deviates from that predicted by simple phase equilibrium theory of coexistence of two gel phases and from discontinuity of the transition. “Discontinuous or Continuous” was a part of the title of Wu and Zhou paper [59]. The authors argue that the transition should be continuous because the gel is composed of distribution of network chains differing in degree of polymerization and the collapse of single linear chains in dilute solutions is molecular weight dependent. This argument in fact objects the application of the mean field theory for the concentrated regime to systems where the transition extends to the region of high swelling degrees of semi-dilute regime. Indeed, an analogous problem was analyzed in the case of phase equilibria in polymer solutions and a correction through “bridging function” was proposed [60,61,62]. Nothing like that has been attempted in the case of gels.

The character of the transition is basically affected by the continuity of network structure causing shear rigidity of the system, which determines the transition from one volume to another one through states of mechanical instabilities. Moreover, due to finite relaxation time of the cross-linked and entangled network, demixing occurs through microphase separation. Microphase separation is characteristic for classical demixing systems, not only for VPT, whenever the concentrated phase is continuous. Already in 1969–1973, Dušek and Sedláček [12,63,64,65] carried out extensive experimental light scattering (LS) study of microphase separation in lightly cross-linked poly(2-hydroxyethyl methacrylate) gels (non-VPT systems) upon change of temperature or solvent exchange. In binary systems, solvent was the minority phase. Upon change of temperature, turbidity developed which was caused by formation of objects (assumedly microdroplets) of size of tens of nanometers (Mie scattering, wavelength ratio method). By keeping the system at constant temperature, the turbidity decreased in time revealing decreasing number of particles while their size remained constant. A liquid macrophase of phase separated solvent was formed outside. The clearing process was completed in 2–3 months for samples about 1-mm thick. The turbidity decay was interpreted by diffusion of the solvent in droplets being under pressure of locally deformed network; the constant size was a result of balance of pressure and interfacial tension on the gel liquid boundary (measured independently). Interestingly, when the cross-linking density was higher, the clearing process was faster and the macrophase equilibrium was reached within hours. The particle size was smaller. Similar experiments with other gels were done later by Style et al. [66] who found that the separated droplets were highly uniform, and their size correlated with the cross-link density. Formation of these droplets is initiated by spinodal decomposition. Ikkai and Shibayama [67] showed that the size of the swollen object must exceed a certain limit in order phase separation to occur in the microform.

In VPT systems, the passage through the region of thermodynamic instability is similar, but due to the extent and steepness of the volume change, it is the concentrated microphase that separates first as minority phase after the VPT threshold is reached. When the concentrated phase becomes continuous, the microphase separated systems transforms to droplet type characterized by slow volume decrease. When deswelling (collapse) transition is observed visually; one sees first a swollen gel to shrink remaining transparent; then turbidity develops, but shrinkage is still relatively fast, followed by the third stage of very slow volume decrease of turbid gel [68,69]. Sometimes, the transition is accompanied by transient formation of mechanical instabilities such as buckling or wrinkling especially when the opposing forces are large (e.g., ionization vs. cross-links) and slow microdroplet stage can be avoided [70]. The dynamics of VPT in gels was described many times both experimentally and theoretically (cf., e.g., [56,57,58,71,72,73,74,75]). Thus, slow relaxation can also be a reason for softening the abruptness of VPT. The best experimental way to approach the abruptness and to find the composition of conjugated phases is to let a series of samples swell until equilibrium at different temperatures and narrow the transition interval (cf., e.g., [76,77]). Monitoring of the transition of the swollen gel phase into the concentrated one by NMR is a very convenient method [78]. Fine resolution spectra characterize the dilute phase while only a broad band is displayed by the concentrated phase.

## 7. Conclusions

Many VPT systems of various chemical compositions are known and used at present time. The chemical composition of the gel can be fine-tuned by examining the effect of the changes in the position and width of the transition by using the equilibrium theory and concentration and temperature dependent interaction function. The present level of theoretical description of VPT dynamics and stress/strain effects seems to be sufficient for design and manufacture of multifunctional gel constructs and devices where slow transition modes can be reduced by miniaturization. Such structurally complex systems will be manufactured by reactive 3D printing controlled by FEM-based programs.
